# Supplementary material for: Dual functions of Aire CARD multimerization in the transcriptional regulation of T cell tolerance
Source: Nat Commun. 2020 Apr 2;11:1625. doi: 10.1038/s41467-020-15448-w (PMC7118133; doi:10.1038/s41467-020-15448-w)
Supplement: Supplementary file 1 — Supplementary Information [file 41467_2020_15448_MOESM1_ESM.pdf]

Dual functions of Aire CARD multimerization in the transcriptional regulation of T cell tolerance

Huoh et al.

Supplementary Information

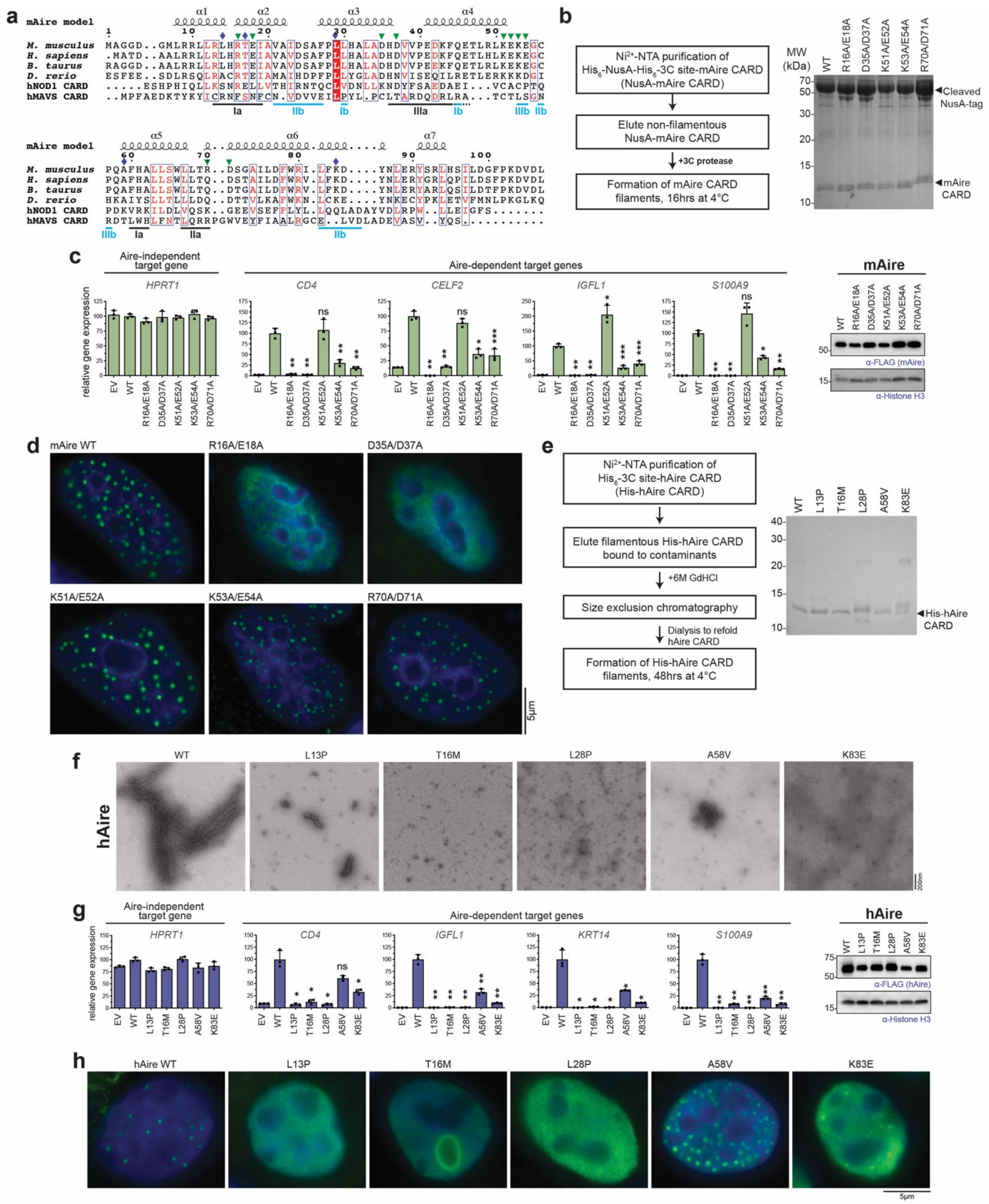

Supplementary Figure 1: CARD filament formation mediates murine and human Aire nuclear foci formation and transcriptional activity.

a. Sequence alignment of Aire CARD domains from various species against human NOD1 (hNOD1) and human MAVS (hMAVS) CARD generated with PROMALS3D and Esprit 3.

The predicted secondary structure for the mAire CARD (based on the homology model in Fig. 1e) is shown on the top. The indicated residue numbers are based on mAire sequence. Green triangles indicate the locations of mAire mutations used in (b-d) and Fig. 1f-h. Blue diamonds indicate the APS-1 patient mutations analyzed in (e-h) and Fig. 1i, j. Conserved CARD-CARD interfaces identified for hNOD1 and hMAVS CARDs are shown at the bottom. Note that Ia, IIa and IIIa surfaces interact with Ib, IIb and IIIb, respectively. Dotted line for IIIa indicates shifted position in the predicted α-helix in Aire CARD relative to hNOD1 and hMAVS CARD.

b. Purification of mAire CARD. Left, schematic of the protein purification protocol; mAire CARD was expressed as a NusA fusion in *E. coli* and was purified by Ni-NTA affinity purification. 3C protease was used to release CARD from the fusion construct. Right, SDS-PAGE gel of 3C protease-cleaved WT and mutant mAire CARD used in EM imaging of filaments in Fig. 1b, f. Note that the NusA tag alone does not form filaments.
c. Transcriptional activity of WT mAire CARD and the mutants in 4D6 cells, as measured by the relative mRNA levels of Aire-dependent genes (represented by *CD4*, *CELF2*, *IGFL1* and *S100A9*). The relative mRNA level of an Aire-independent gene, *HPRT1*, is shown as a negative control. Data are presented as mean ± s.d., $n = 3$. P-values (two-tailed t-test) were calculated in comparison to WT mAire. * $p < 0.05$; ** $p < 0.01$; $p > 0.05$ is not significant (ns). Exact p-values are provided in the Source Data File. Right, western blot (WB) showing the expression levels of FLAG-tagged mAire.
d. Representative fluorescence microscopy images of WT and mAire mutants in 4D6 cells.
e. Purification of hAire CARD. Left, schematic of the protein purification protocol. Unlike mAire CARD, hAire CARD fused to NusA-tag yielded very low levels of purified protein. Successful purification of hAire CARD from *E. coli* required a minimal N-terminal His_6 tag and a refolding step using 6 M guanidine hydrochloride (GdHCl). 3C protease was used to remove the His_6 tag. Right, SDS-PAGE gel of 3C protease-cleaved WT and mutant hAire CARD used in (f).
f. Representative EM images of WT and hAire CARD with APS-1 patient mutations indicated in (a).
g. Transcriptional activity of WT and mutant hAire in 293T cells. Experiments were performed as in (c) and are presented as mean ± s.d., $n = 3$. P-values (two-tailed t-test) were calculated in comparison to WT hAire. * $p < 0.05$; ** $p < 0.01$; $p > 0.05$ is not significant (ns). Exact p-values are provided in the Source Data File.
h. Representative fluorescence microscopy images of FLAG-tagged hAire in 293T using anti-FLAG.

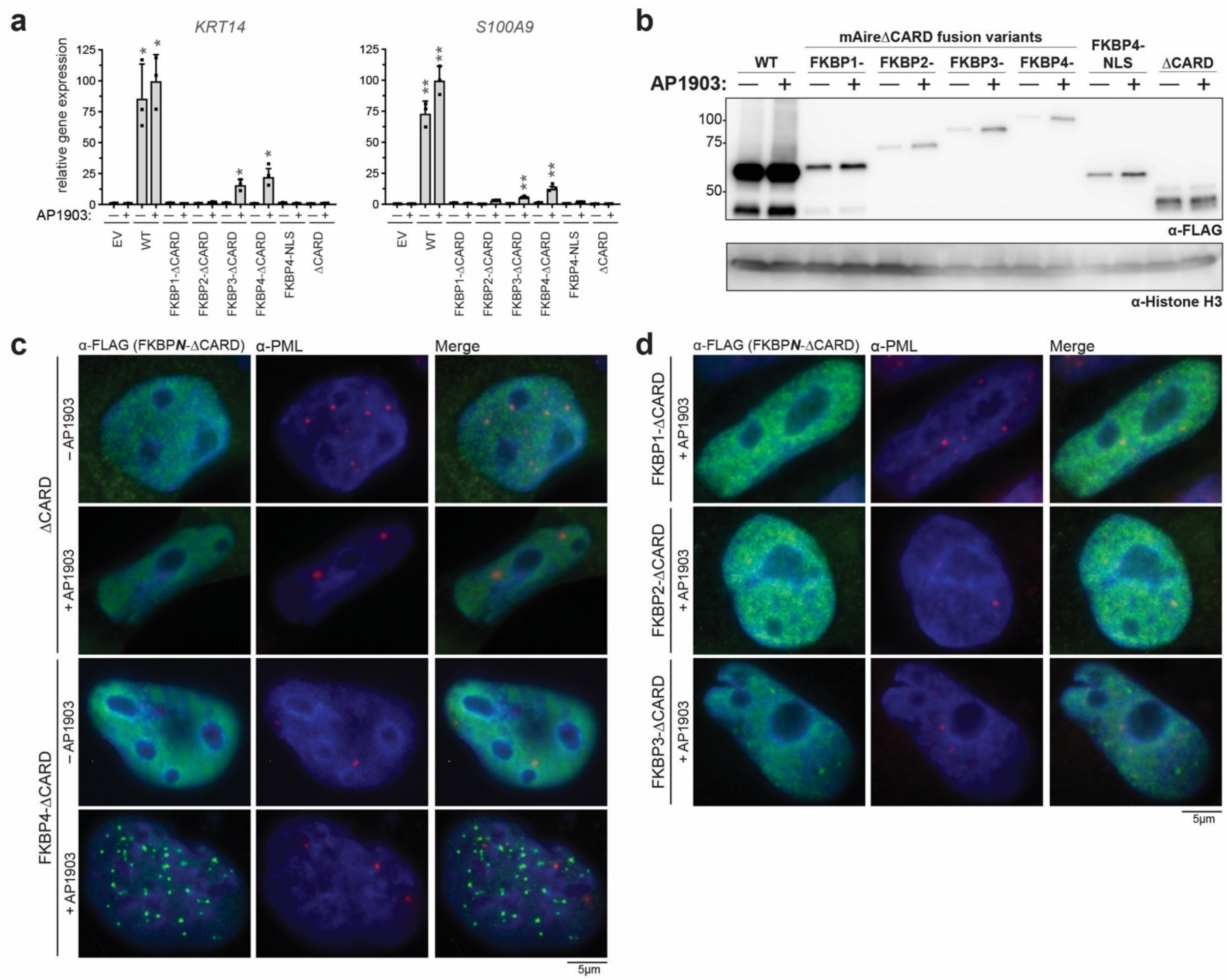

Supplementary Figure 2: Chemically induced multimerization partially restores the transcriptional activity of AireΔCARD.

a. Transcriptional activity of ΔCARD fused with tandem repeats of FKBP (FKBP1-4) in the presence and absence of chemical dimerizer (AP1903) in 293T cells. Experiments were performed as in Fig. 1g and are presented as mean ± s.d., $n = 3$. Note that *KRT14* and *S100A9* are Aire-dependent genes. P-values (two-tailed t-test) were calculated in comparison to empty vector (EV) + AP1903. * $p < 0.05$; ** $p < 0.01$. Exact p-values are provided in the Source Data File.

b. WB showing the expression levels of the proteins used in (a) and Fig. 2b.

c.-d. Representative fluorescence microscopy images of mAire ΔCARD fusion variants in 4D6 cells. Cells were treated with DMSO or 5 μm AP1903 for 24 hours prior to fixation. Cells were immunostained with anti-FLAG (mAire variants) and anti-PML. Images of anti-FLAG immunostained cells are reproduced from Fig. 2c, d.

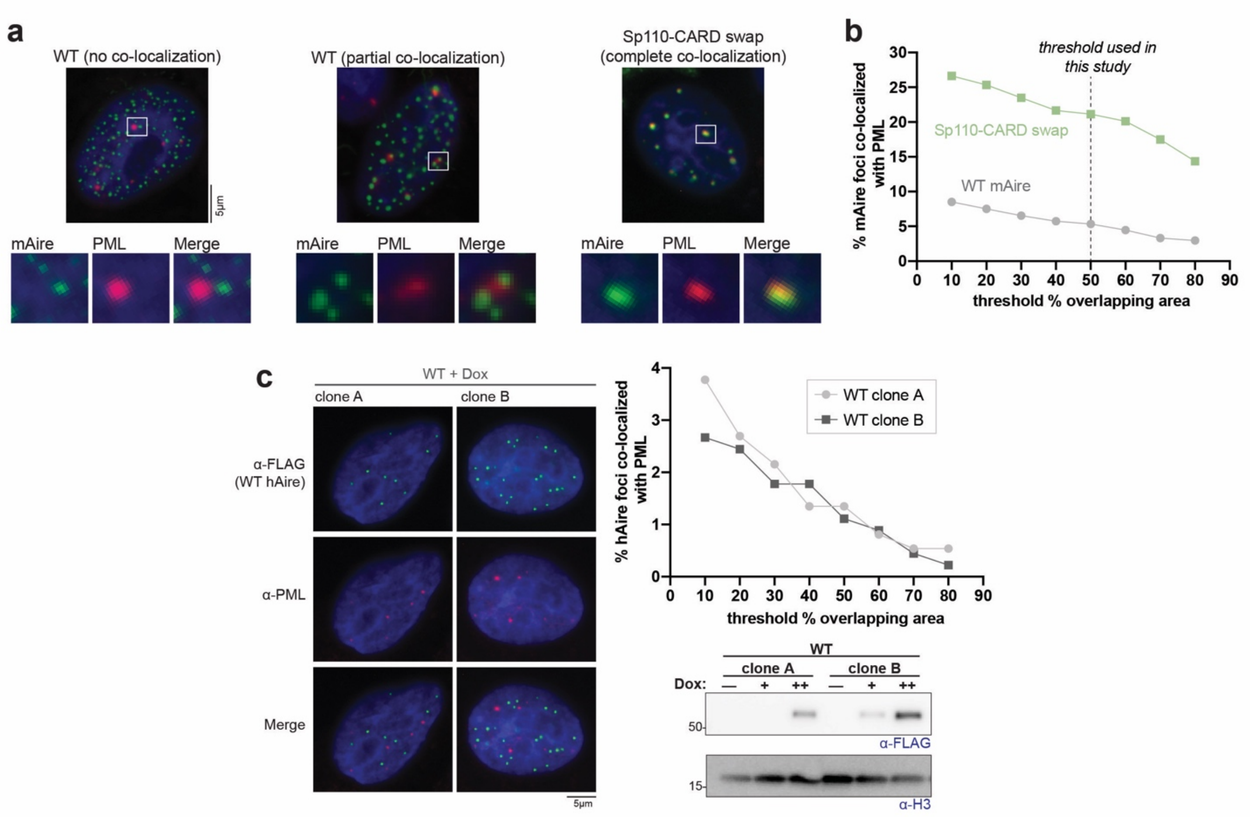

Supplementary Figure 3: Quantitative analysis of PML body co-localization.

a. Examples of Aire foci that have no overlap, partial overlap, and complete overlap with endogenous PML bodies. We observed that some WT and Sp110-CARD swap foci were not completely overlapping with PML, but rather positioned adjacent to PML bodies. For systematic and quantitative analysis of the degree of co-localization of Aire foci and PML bodies, see (b).
b. Fraction of Aire (WT Aire or Sp110-CARD swap) foci co-localized with PML bodies while defining co-localization as having greater than a certain threshold % overlapping area (x-axis). See Methods for details of automated image analysis workflow. Sp110-CARD swap foci are associated with PML bodies significantly more than WT mAire regardless of the choice of threshold. A total of 2022 and 383 Aire foci were examined for WT mAire and Sp110-CARD swap samples, respectively. For quantitation of Aire foci co-localized with PML, we arbitrarily chose the threshold definition of 50% overlap area.
c. Representative fluorescence microscopy images of two independent monoclonal 4D6 cells stably expressing WT hAire-FLAG under a doxycycline-inducible promoter. 4D6 cells were induced at 1 μg/ml doxycycline for 24 hrs before immunostaining with anti-FLAG and anti-PML. Top right, quantification of Aire foci co-localized with PML bodies from the two clones of 4D6 cells stably expressing WT hAire-FLAG. Analysis was done as in (b) and shows minimal co-localization of WT hAire with PML bodies, independent of the choice of the threshold definition. Statistical significance comparison between WT hAire-FLAG foci overlapping with PML (50% threshold) from the two 4D6 clones was calculated using a two-tailed Student's t-test (see Methods for details) and determined to be not significant (p =

0.9118). A total of 371 and 450 Aire foci were examined for WT clones A and B, respectively. Bottom right, WB analysis of WT hAire-FLAG expression in the presence of 0.2 μg/ml (+) or 1 μg/ml (++) of doxycycline.

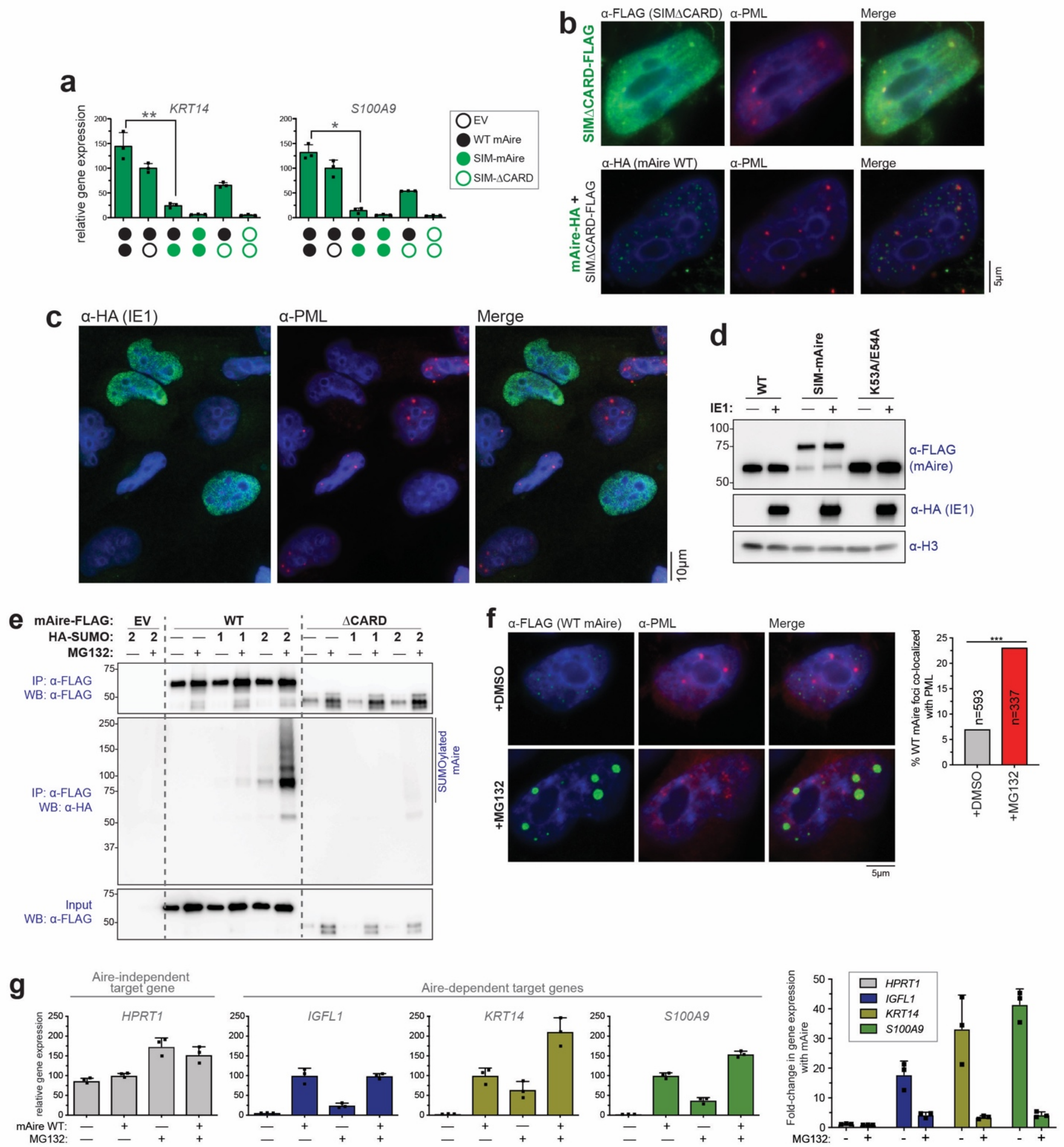

Supplementary Figure 4: Effect of SIM-mAire, IE1 and MG132 on Aire foci localization.

a. Transcriptional activity of WT mAire (black circle) with and without co-expression of SIM-mAire (green circle) in 293T cells. Each circle represents 0.6 µg/ml DNA transfected. Experiments were performed as in Fig. 4d and presented as mean ± s.d., *n* = 3. Experiments were performed as in Fig. 1g and are presented as mean ± s.d., *n* = 3. P-values (two-tailed t-test) were calculated in comparison to WT mAire. ** p = 0.0015 and * p = 0.016. Note that *KRT14* and *S100A9* are Aire-dependent target genes.

b. Representative fluorescence microscopy images of SIMΔCARD-FLAG with or without co-expression of WT mAire-HA in 4D6 cells.
c. Representative fluorescence microscopy images of IE1-HA in 4D6 cells. Cells that express IE1 show diffuse nuclear staining of endogenous PML, whereas cells with no IE1 expression have intact PML bodies.
d. WB showing the expression levels of the proteins used in Fig. 4e, f.
e. SUMO modification analysis of FLAG-tagged WT mAire and ΔCARD. mAire (0.6 μg/ml DNA) was co-expressed with HA-SUMO1 or -SUMO2 (0.6 μg/ml DNA) in 293T cells and the immunoprecipitation was performed as in Fig. 3c.
f. Representative fluorescence microscopy images of FLAG-tagged WT mAire in the presence and absence of MG132 (10 μM). 4D6 cells were treated with MG132 for 4 hrs before fixation. Right, quantitative analysis of Aire foci co-localized with PML bodies. n = number of Aire foci examined per sample. Statistical significance comparison was calculated using a two-tailed Student's t-test for two population proportions where each population consists of all individual Aire foci examined.*** p = 2.159e-12.
g. Transcriptional activity of WT mAire in the presence and absence of MG132 (10 μM). Cells were treated with MG132 for 16 hrs. The fold-change as a result of Aire expression was plotted in the right panel. Experiments were performed as in Fig. 1g and are presented as mean ± s.d., $n = 3$.

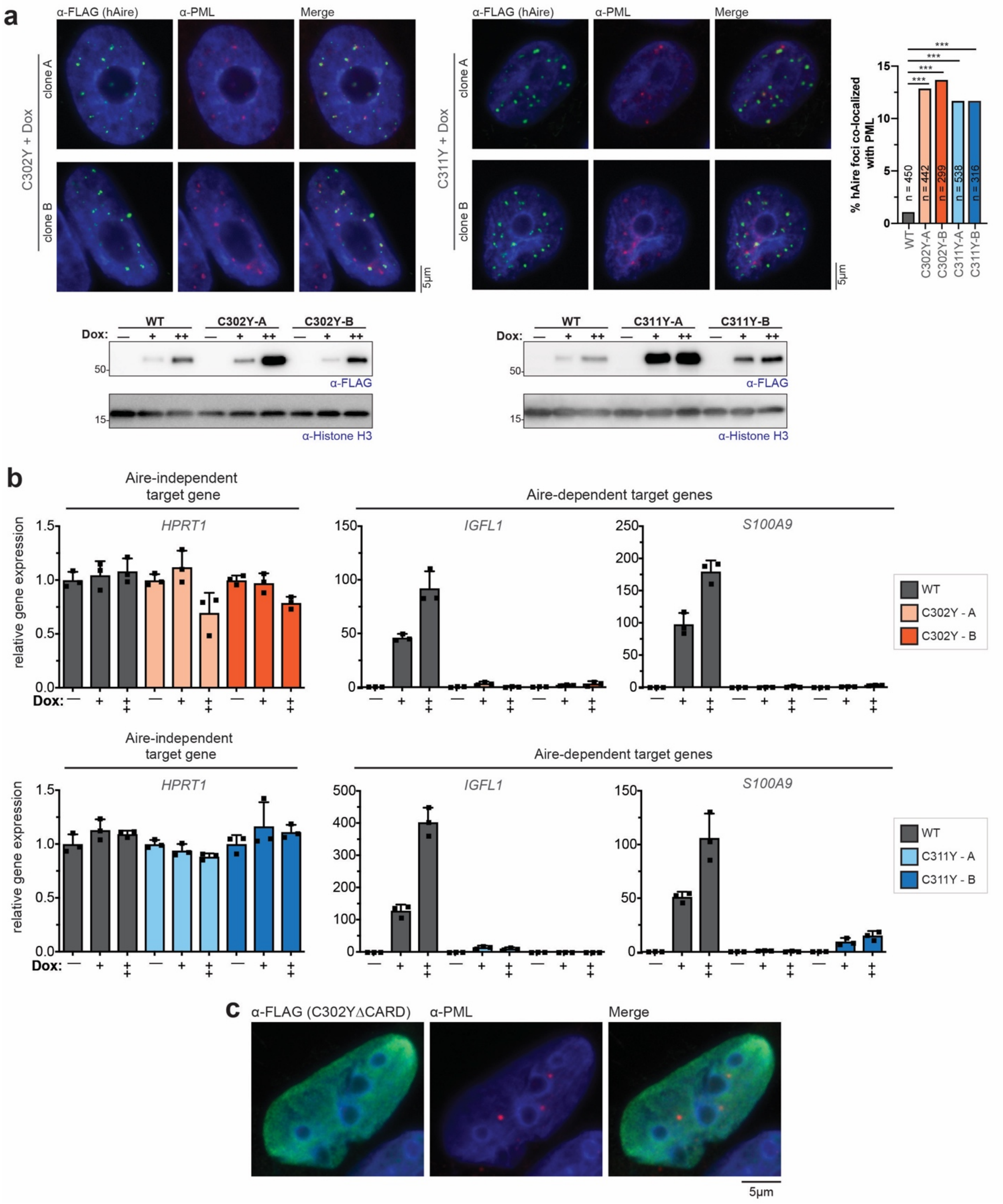

Supplementary Figure 5: C302Y and C311Y mutations increase PML-association of hAire foci.

a. Representative fluorescence microscopy images of 4D6 cells stably expressing C302Y or C311Y hAire-FLAG under a doxycycline-inducible promoter. Two independent monoclonal

cell lines for each hAire variant are shown. Cells were induced at 1 μg/ml doxycycline for 24 hrs before immunostaining with anti-FLAG and anti-PML. Right, quantitation of WT or mutant Aire foci co-localized with PML bodies. n = number of Aire foci examined per sample is indicated. Statistical significance comparisons were calculated using a two-tailed Student's t-test for two population proportions where each population consists of all individual Aire foci examined. *** $p < 0.001$; $p > 0.05$ is not significant (ns). Exact p-values are provided in the Source Data File. For WT cells, clone B from Supplementary Fig. 3c was used. See Supplementary Fig. 3c for representative fluorescence microscopy images and comparison of two WT clones. Bottom, western blots showing the expression levels of hAire WT and variants. For clarity, the bottom right panel of WT hAire-FLAG western blots was reproduced from Supplementary Fig. 3c.

b. Transcriptional activity of hAire WT, C302Y (top panel), and C311Y (bottom panel) stably expressed in 4D6 cells in the presence of 0.2 μg/ml (+) and 1 μg/ml (++) of doxycycline for 48 hrs. Cells were harvested and RT-qPCR was performed as in Fig. 1g. Transcriptional activity was normalized to samples without doxycycline. Data are presented as mean ± s.d., *n* = 3. Note that *IGFL1* and *S100A9* are Aire-dependent genes and the Aire-independent gene *HPRT1* is shown as a negative control.
c. Representative fluorescence microscopy images of FLAG-tagged hAireΔCARD C302Y variant (C302YΔCARD) in 4D6 cells. Cells were immunostained with anti-FLAG and anti-PML. C302YΔCARD does not form nuclear foci.

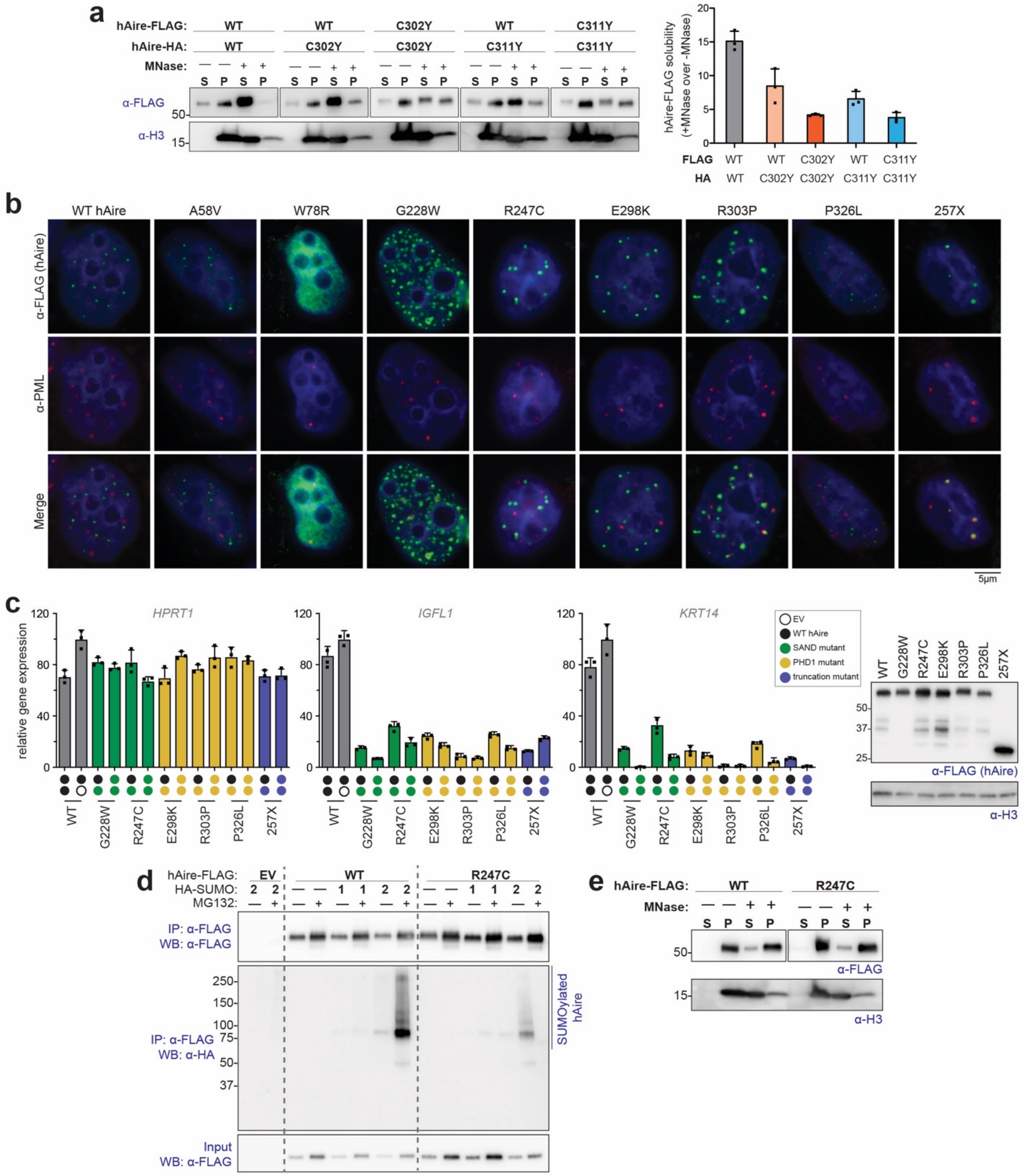

Supplementary Figure 6: PML body-localization is frequently, but not always, associated with dominant negative mutants.

a. Chromatin fractionation analysis of FLAG-tagged WT hAire with and without co-expression with HA-tagged C302Y and C311Y as described in Fig. 3d. Right, fold-increase in WT hAire solubility upon MNase treatment is plotted. WT hAire becomes more MNase-

insensitive when co-expressed with C302Y or C311Y. Western blot quantification was performed in ImageJ v. 2.0.0 and are presented as mean ± s.d., *n* = 3.

b. Representative fluorescence microscopy images of FLAG-tagged hAire WT and APS-1 mutants expressed in 4D6 cells. For clarity, images of WT hAire were reproduced from Fig. 6c. See Table 1 for the summary of all APS-1 mutations tested in this study.
c. Transcriptional activity of hAire WT (black circle) and APS-1 patient mutants (colored circles) either individually or in combination, in 293T cells. Empty vector (EV) is indicated with empty circle. Each circle represents 0.5 μg/ml DNA transfected. Data are presented as mean ± s.d., *n* = 3. See Table 1 for the summary of all APS-1 mutations tested in this study.
d. SUMOylation analysis of hAire WT and R247C. Experiments were performed as in Fig. 3c. The mutant shows a decreased level of SUMO2 modification.
e. Chromatin fractionation analysis of hAire WT and R247C. Experiments were performed as in Fig. 3d. The mutant shows a comparable level of MNase-sensitivity as WT hAire.

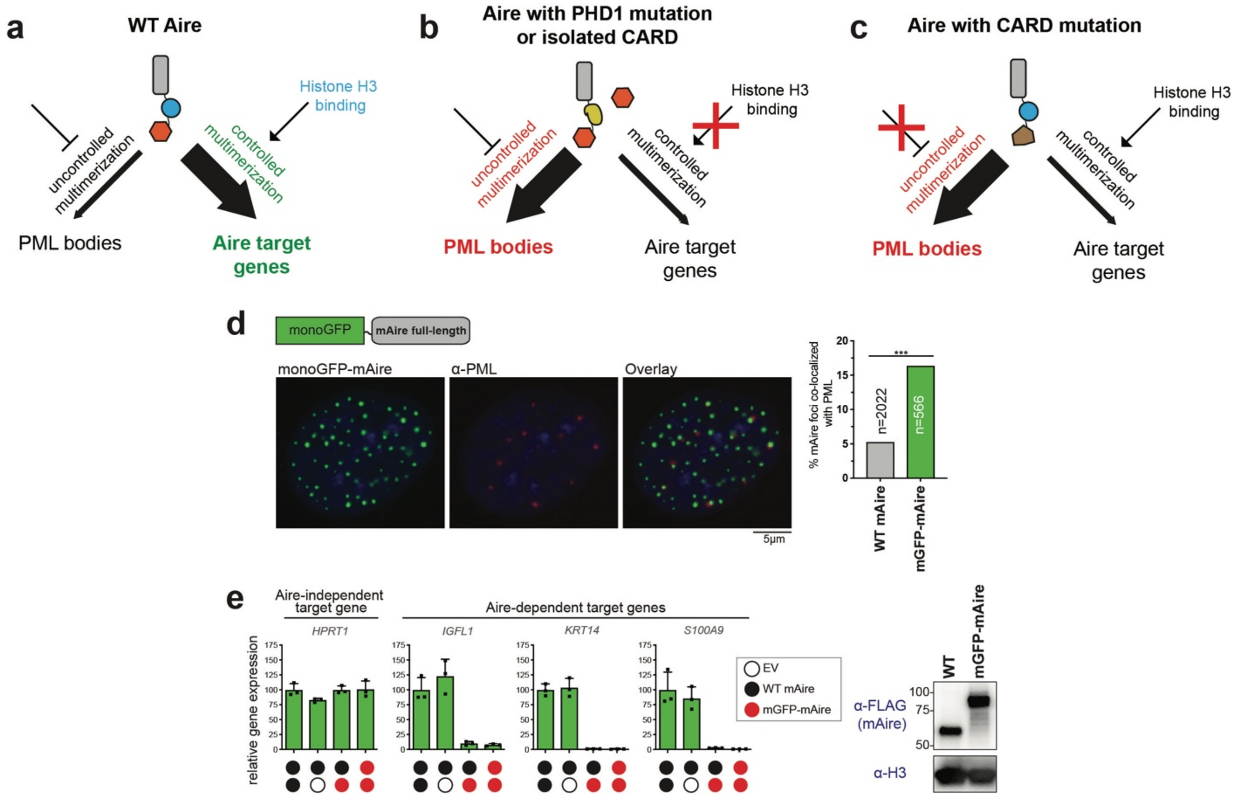

Supplementary Figure 7: Uncontrolled vs. controlled multimerization of Aire variants determines nuclear localization.

a-c. Correct localization of Aire may require controlled polymerization of CARD at the right place and the right time. WT Aire CARD may be normally suppressed (but not completely prevented) from uncontrolled multimerization until it forms appropriate interactions with target chromatin sites via PHD1 (a). However, defect in PHD1 may prevent controlled multimerization, tilting the balance in favor of uncontrolled multimerization and PML localization (b). CARD mutations may also lead to PML localization, if the mutations impair the mechanism for suppressing uncontrolled multimerization (c). Using this model, we can explain how chemically induced homo-polymer of FKBP4-ΔCARD evades PML localization and is transcriptionally active. The addition of a chemical dimerizer to induce multimerization may mimic controlled multimerization of WT as it provides the C-terminal portion of Aire sufficient time to form interactions with target chromatin before inducing multimerization. Furthermore, this model can reconcile the loss of activity of Sp110-CARD swap (Fig. 3a) or N-terminal GFP fusion construct [to be discussed below in (d-e)], as these modifications in or near Aire CARD may interfere with the suppression mechanism of uncontrolled CARD multimerization.

d. Representative fluorescence microscopy images of mAire WT fused with monomeric GFP (mGFP) in 4D6 cells. Quantitative analysis (right) shows that mGFP fusion increases PML co-localization of mAire. n = number of Aire foci examined per sample is indicated. Statistical significance comparisons were calculated using a two-tailed Student's t-test for two population proportions where each population consists of all individual Aire foci examined. *** $p = 2.952e-18$.

e. Transcriptional activity of WT mAire (black circle) and mGFP-mAire (red circle), either individually or in combination, in 293T cells. Empty vector (EV) is indicated with empty circle. Each circle represents 0.6 μg/ml DNA transfected. Data are presented as mean ± s.d., $n = 3$. Right, WB showing expression levels of the proteins used in this study. mGFP-mAire is not only defective in transcriptional activity, but exerts a dominant negative effect on co-expressed WT Aire.

Supplementary Table 1: PCR primers used for generating expression vectors in this study

Primer Name	Sequence (5' to 3')
mAire-R16A-E18A	CTGCTGAGGCTGCACGCCACCGCGATCGC GGTGGCCATAG
mAire-D35A-D37A	CTGCATGCTCTAGCCGCCCACGCCGTGGT CCCTGAGGAC
mAire-K51A-E52A	GGAGACGCTCCGTCTGGCGGCGAAGGAA GGCTGCCC
mAire-K53A-E54A	CGCTCCGTCTGAAGGAGGCGGCAGGCTGC CCCCAGGC
mAire-R70A-D71A	GTCCTGGCTCCTGACCGCGGCCAGTGGGG CCATCCTG
5' mAIRE-D104L-HindIII	GCATCCTGGACGGCTTCCCAAAGCTTGTG GACCTAAACCAGTCCC
hAIRE_L13P_For	ACGCCGGCTTCTGAGGCCGCACCGCACGG AGATCG
hAIRE_T16M_For	TCTGAGGCTGCACCGCATGGAGATCGCGG TGGCC
hAIRE_L28P_For	GGACAGCGCCTTCCCACCGCTGCACGCGC TGGCTG
hAIRE_A58V_for	GAGGGCTGCCCCCAGGTCTTCCACGCCCT CCTGTC
hAIRE_K83E_for	CTGGACTTCTGGAGGGTGCTGTTCGAGGA CTACAACCTGGAGCGCTATG
hAIRE_R247Cfor	GTGGGAAGAACAAGGCCTGCAGCAGCAG TGGCCCG
hAIRE_G228Wfor	GTGCATCCAGGTTGGCTGGGAGTTCTACA CTCCCAGC
huAIRE_C302Y_for	GGACGAGTGTGCCGTGTATCGGGACGGCG GGGA
huAIRE_R303P_for	ACGAGTGTGCCGTGTGTCCGGACGGCGGG GAGCTC
huAIRE_C311Y_for	CGGGGAGCTCATCTGCTATGACGGCTGCC CTCG
huAIRE_P326L_for	GCCTGCCTGTCCCCTCTGCTCCGGGAGAT CCCC
AmpFor	CCATGAGTGATAACACTGCGGCCAACTTA CTTCTGAC
KanRev	CTCGTCCAACATCAATACAACCTATTAAT TTCCCCTCGTC
NotI-Kozak-mAire_startFOR	ATATGCGGCCGCCACCATGGCAGGTGGGG ATGGAATG
hAire_end_FLAGstop_AscIREV	ATATGGCGCGCCTCACTTGTCGTCATCAT CCTTGTAATCGGAGGGGAAGGGGGC

KpnI_ hAIRE1for	CGCGGTACCAGATGGCGACGGACGCGGCGC
hAIRE105stopAvrIIrev	CGCCCTAGGTCAGTCCACATCTTTGGGGAAGCTG
5KpnI-mAIRE	ATATGGTACCAGATGGCAGGTGGGGATGGAATG
mAIRE106stopAvrIIrev	CGCCCTAGGTCAGTCCACATCTTTTGGGAAGCCG
BglII_mAIRE107ATGfor	ATATAGATCTGATGCTAAACCAGTCCCGGAAAGGG
mAIRE174FLAGrev	CTTGTCGTCATCGTCTTTGTAGTCTGACTCCAAGTTGCCATCTGGC
KpnI_mAIRE107For	ATATGGTACCAGCTAAACCAGTCCCGGAAAGG
mAIRE173_3XFLAGA_REV	GTCGTGGTCCTTGTAGTCACCGTCGTGGTCCTTGTAGTCTGACTCCAAGTTGCCATCTGG
mAIRE173_3XFLAGB_TEV1REV	AAGATTCTCCTTGTCGTCGTCGTCCTTGTAGTCGATGTCGTGGTCCTTGTAGTCACC
mAIRE173_3XFLAGB_TEV2Age1REV	CGCGACCGGTCCCTGAAAATAAAGATTCTCCTTGTCGTCATC
pEGFPN1A206K	CCTGAGCACCCAGTCCAAGCTGAGCAAAGACCCCAAC
NheI_START_hAire106_For	ATATGCTAGCATGCTCAGCCAGCCCCGGAAGGGG
hAireR257_FLAGstop_AscI_REV	ATATGGCGCGCCTTACTTGTCATCGTCGTCCTTGTAGTCAACCAGAGGCTTCGGGC
3HindIII-FKBP1-2-nostop	ATATAAGCTTCTGCACGCCCTCGACTT
5NheI_startFKBP3_forgBlock	ATATGCTAGCATGCAGCCTCGGCTAGAAGGC
3HindIII_FKBP3_forgBlock	ATATAAGCTTCAGTGTGCTGGAGTTCAGGGATTG
HindIII_FKBP3gblock_For	ATATAAGCTTCAGCCTCGGCTAGAAG
NheI_startRNF4aa38_FOR	ATATGCTAGCATGGAGATCTCCTTGGAAGCAGAA
RNF4aa129_BglII_REV	ATATAGATCTAGTACCTGAGGGCCTGAGG

Supplementary Table 2: qPCR primers used in this study

Primer Name	Sequence (5' to 3')
CD4-FW	GGACAGGTCCTGCTGGAATC
CD4-RV	CAATGAAAAGCAGGAGGCCG
CELF2-FW	TCCTTGACCTCTCTCGGGAC
CELF2-RV	AAGTCCTCCATTCAGAGCCG
GAPDH-FW	TGATGACATCAAGAAGGTGGTGAAG
GAPDH-RV	TCCTTGGAGGCCATGTGGGCCAT
HPRT1-FW	TGAAGAGCTATTGTAATGACCAGTCAAC
HPRT1-RV	AGCAAGCTTGCGACCTTGACCA
IGFL1-FW	CTCCCCGAGGCTGCATCGTA
IGFL1-RV	TGGCACAGCATCAGGTAAGGA
KRT14-FW	CAGTCCCTACTTCAAGACCATTGA
KRT14-RV	ACTGTGGCTGTGAGAATCTTGTTC
S100A9-FW	CTGGTGCGAAAAGATCTGCA
S100A9-RV	CCTTTTCATTCTTATTCTCCTTCTTGAG
