# Supplementary material for: Dual functions of Aire CARD multimerization in the transcriptional regulation of T cell tolerance
Source: Nat Commun. 2020 Apr 2;11:1625. doi: 10.1038/s41467-020-15448-w (PMC7118133; doi:10.1038/s41467-020-15448-w)
Supplement: Supplementary file 3 — Reporting Summary [file 41467_2020_15448_MOESM3_ESM.pdf]

natureresearch

Corresponding author(s): Sun Hur

Last updated by author(s): Mar 5, 2020

Reporting Summary

Nature Research wishes to improve the reproducibility of the work that we publish. This form provides structure for consistency and transparency in reporting. For further information on Nature Research policies, see Authors & Referees and the Editorial Policy Checklist.

Statistics

For all statistical analyses, confirm that the following items are present in the figure legend, table legend, main text, or Methods section.

n/a	Confirmed	
☐	☒	The exact sample size (*n*) for each experimental group/condition, given as a discrete number and unit of measurement
☐	☒	A statement on whether measurements were taken from distinct samples or whether the same sample was measured repeatedly
☐	☒	The statistical test(s) used AND whether they are one- or two-sided<br>*Only common tests should be described solely by name; describe more complex techniques in the Methods section.*
☒	☐	A description of all covariates tested
☒	☐	A description of any assumptions or corrections, such as tests of normality and adjustment for multiple comparisons
☐	☒	A full description of the statistical parameters including central tendency (e.g. means) or other basic estimates (e.g. regression coefficient) AND variation (e.g. standard deviation) or associated estimates of uncertainty (e.g. confidence intervals)
☐	☒	For null hypothesis testing, the test statistic (e.g. *F*, *t*, *r*) with confidence intervals, effect sizes, degrees of freedom and *P* value noted<br>*Give P values as exact values whenever suitable.*
☒	☐	For Bayesian analysis, information on the choice of priors and Markov chain Monte Carlo settings
☒	☐	For hierarchical and complex designs, identification of the appropriate level for tests and full reporting of outcomes
☒	☐	Estimates of effect sizes (e.g. Cohen's *d*, Pearson's *r*), indicating how they were calculated

Our web collection on statistics for biologists contains articles on many of the points above.

Software and code

Policy information about availability of computer code

Data collection	Immunofluorescence microscopy data was collected on Zeiss Axio Imager M1 fluorescence microscope using the acquisition software Slidebook 4.2. qPCR data was collected on CFX-Connect detection system with Bio-Rad CFX manager 3.1 software.
Data analysis	Negative stained electron micrographs were processed in EMAN2 and RELION 3.0. ImageJ v2.0.0 was used for the quantification of western blot images. Phyre2 and FUGUE 2.01 were used for structure homology modeling. PROMALS3D and ESPript 3 were used to make the structure-based sequence alignment. An in-house Matlab version R2018b program was used to analyze immunofluorescence microscopy images and is available upon request.

For manuscripts utilizing custom algorithms or software that are central to the research but not yet described in published literature, software must be made available to editors/reviewers. We strongly encourage code deposition in a community repository (e.g. GitHub). See the Nature Research guidelines for submitting code & software for further information.

Data

Policy information about availability of data

All manuscripts must include a data availability statement. This statement should provide the following information, where applicable:

- Accession codes, unique identifiers, or web links for publicly available datasets
- A list of figures that have associated raw data
- A description of any restrictions on data availability

The source data underlying Table 1, Figures 1c, d, g, h; 2b; 3a-c, e; 4b-d, f; 5b, d; 6b-e; 7a, b, d and Supplementary Figures 1b, c, e, g; 2a, b; 3b, c; 4a, d-g; 5a, b; 6a, c-e and 7d, e are provided as a Source Data file. All other data are included in the Supplementary Information or available from the authors upon reasonable requests.

Field-specific reporting

Please select the one below that is the best fit for your research. If you are not sure, read the appropriate sections before making your selection.

☒ Life sciences ☐ Behavioural & social sciences ☐ Ecological, evolutionary & environmental sciences

For a reference copy of the document with all sections, see nature.com/documents/nr-reporting-summary-flat.pdf

Life sciences study design

All studies must disclose on these points even when the disclosure is negative.

Sample size	The number of mammalian tissue culture cells used for chromatin fractionation and pull-down experiments were determined by the amount needed to visualize proteins of interest for western blotting. For quantitative PCR experiments, the amount of tissue culture cells used was dependent on the quantity of total RNA that could be extracted from the cells. Sample size for quantified immunofluorescence microscopy experiments was not predetermined. When writing the in-house MATLAB program for quantitative analysis, we kept the sample size low; once we processed the entire data sets, we saw that qualitatively the results were the same even when only a small fraction of the data (~40 foci) was examined. See Figure legends for details.
Data exclusions	For immunofluorescence microscopy analysis, our program excluded cells that did not have both expression of Aire and PML along with foci that were dimmer than background levels. Please see Methods for details of our in-house Matlab program.
Replication	All biochemical assays, pull-downs, and quantitative PCR experiments were performed 2-4 individual times. For quantitative PCR experiments, in addition to 2-3 biological replicates, we included 3 technical replicates for each experiment. For immunofluorescence microscopy experiments, any experiment with quantification had 2-4 biological replicates. Only experimental data that were successfully replicated in all attempts are reported. See Figure legends for details.
Randomization	For immunofluorescence microscopy, images were taken at random locations on the cover slip. For electron microscopy, images were also taken at random locations throughout the grid. Other experiments in this study were not subjected to randomization as the identity of the samples are predetermined during experiments; the experimental results would not be interpretable if these samples were randomized.
Blinding	When performing experiments, there were no samples that could be blinded as the identity of the samples are predetermined during experiments. When writing the in-house MATLAB program to perform quantification analyses on immunofluorescence microscopy images, the program code author was blinded by the identity of the sample sets and unaware of our hypotheses to ensure the code was written to be unbiased.

Reporting for specific materials, systems and methods

We require information from authors about some types of materials, experimental systems and methods used in many studies. Here, indicate whether each material, system or method listed is relevant to your study. If you are not sure if a list item applies to your research, read the appropriate section before selecting a response.

Materials & experimental systems

n/a	Involved in the study	
☐	☒	Antibodies
☐	☒	Eukaryotic cell lines
☒	☐	Palaeontology
☒	☐	Animals and other organisms
☒	☐	Human research participants
☒	☐	Clinical data

Methods

n/a	Involved in the study	
☒	☐	ChIP-seq
☒	☐	Flow cytometry
☒	☐	MRI-based neuroimaging

Antibodies

Antibodies used	mouse anti-FLAG M2 (Sigma-Aldrich Cat. No. F3165); rabbit anti-PML H238 (Santa Cruz Biotechnology Cat. No. sc5621); Cy5-, Alexa647-, or Alex488-conjugated anti-mouse IgG (Jackson ImmunoResearch Cat. No. 715-175-151, 715-605-151, 715-545-151, respectively); Cy3-, Cy5-, FITC-, or Alexa488-conjugated anti-rabbit IgG (Jackson ImmunoResearch Cat. No. 711-165-152, 711-175-152, 711-095-152, 711-545-152, respectively), mouse anti-FLAG HRP M2 (Sigma-Aldrich Cat. No. A8592), mouse anti-histone H3 1B1B2 (Cell Signaling Technology Cat. No.14269S), rabbit anti-HA C29F4 (Cell Signaling Technology Cat. No. 3724S), mouse anti-HA 6E2 (Cell Signaling Technology Cat. No. 2367S), anti-rabbit IgG-HRP (Cell Signaling Technology Cat. No. 7074S), anti-mouse IgG-HRP (GE Healthcare Cat. No. NA931V).
Validation	All primary antibodies were validated previously by the manufacturer. Citations of studies using these antibodies and user ratings are provided on the manufacturer's websites: mouse anti-FLAG M2 (https://www.sigmaaldrich.com/catalog/product/sigma/f3165); rabbit anti-PML H238 (https://www.scbt.com/p/pml-antibody-h-238); Cy5-, Alexa647-, or Alex488-conjugated anti-mouse IgG (https://www.jacksonimmuno.com/catalog/products); Cy3-, Cy5-, FITC-, or Alexa488-conjugated anti-rabbit IgG

(https://www.jacksonimmuno.com/catalog/products), mouse anti-FLAG HRP M2 (https://www.sigmaaldrich.com/catalog/product/sigma/a8592), mouse anti-histone H3 1B1B2 (https://www.cellsignal.com/products/primary-antibodies/histone-h3-1b1b2-mouse-mab/14269), rabbit anti-HA C29F4 (https://www.cellsignal.com/products/primary-antibodies/ha-tag-c29f4-rabbit-mab/3724), mouse anti-HA 6E2 (https://www.cellsignal.com/products/primary-antibodies/ha-tag-6e2-mouse-mab/2367), anti-rabbit IgG-HRP (https://www.cellsignal.com/products/secondary-antibodies/anti-rabbit-igg-hrp-linked-antibody/7074), anti-mouse IgG-HRP (https://www.gelifesciences.com/en/us/shop/protein-analysis/blotting-and-detection/blotting-standards-and-reagents/amersham-ecl-hrp-conjugated-antibodies-p-06260).

Eukaryotic cell lines

Policy information about cell lines

Cell line source(s)	Human embryonic kidney cells 293T were a generous gift Dr. Dan Stetson, University of Washington; Seattle, WA. 4D6 cells were originally derived from human thymic epithelium from children undergoing cardiac surgery and provided to the laboratory of Diane Mathis.
Authentication	No form of authentication was used for these cell lines
Mycoplasma contamination	These cells were verified to be mycoplasma free by using the MycoAlert Mycoplasma Detection Kit (Lonza, Cat. No. LT07-318).
Commonly misidentified lines (See ICLAC register)	No commonly misidentified lines were used in this study.
